# Detection of genetic association and functional polymorphisms of *UGDH* affecting milk production trait in Chinese Holstein cattle

**DOI:** 10.1186/1471-2164-13-590

**Published:** 2012-11-02

**Authors:** Qing Xu, Gui Mei, Dongxiao Sun, Qin Zhang, Yuan Zhang, Cengceng Yin, Huiyong Chen, Xiangdong Ding, Jianfeng Liu

**Affiliations:** 1 Department of Animal Genetics and Breeding, College of Animal Science and Technology, Key Laboratory of Animal Genetics and Breeding of Ministry of Agriculture, National Engineering Laboratory for Animal Breeding, China Agricultural University, Beijing 100193, China; 2 Institute of Life Science and Biotechnology, Beijing Jiaotong University, Beijing 100044, China; 3 Institute of Animal Science, Guangdong Academy of Agricultural Sciences; State Key Laboratory of Livestock and Poultry Breeding, Guangzhou 510640, China

**Keywords:** Dairy cattle, BTA6, Positional candidate, Milk traits, *UGDH*, Function validation

## Abstract

**Background:**

We previously localized a quantitative trait locus (QTL) on bovine chromosome 6 affecting milk production traits to a 1.5-Mb region between *BMS483* and *MNB-209* via genome scanning followed by fine mapping.

**Results:**

Totally 15 genes were mapped within such linkage region through bioinformatic analysis of the cattle-human comparative map and bovine genome assembly. Of them, the UDP-glucose dehydrogenase (*UGDH*) was suggested as a potential positional candidate gene for milk production traits based on its corresponding physiological and biochemical functions and genetic effects. By sequencing all the coding exons and the untranslated regions in *UGDH* with pooled DNA of 8 sires represented the separated families detected in our previous studies, a total of ten SNPs were identified and genotyped in 1417 Holstein cows of 8 separation families. Individual SNP-based association analysis revealed 4 significant associations of SNP Ex1-1, SNP Int3-1, SNP Int5-1, and SNP Ex12-3 with milk yield (P < 0.05), and 2 significant associations of SNP Ex1-1 and SNP Ex12-3 with protein yield (P < 0.05). Furthermore, our haplotype-based association analyses indicated that haplotypes G-C-C, formed by SNP Ex12-2-SNP Int11-1-SNP Ex11-1, T-G, formed by SNP Int9-3-SNP Int9-2, and C-C, formed by SNP Int5-1-SNP Int3-1, are significantly associated with _protein percentage_ (F=4.15; P=0.0418) and _fat percentage_ (F=5.18~7.25; P=0.0072~0.0231). Finally, by using an *in vitro* expression assay, we demonstrated that the A allele of SNP Ex1-1 and T allele of SNP Ex11-1of *UGDH* significantly decreases the expression of UGDH by 68.0% at the RNA, and 50.1% at the protein level, suggesting that SNP Ex1-1 and Ex11-1 represent two functional polymorphisms affecting expression of *UGDH* and may partly contributed to the observed association of the gene with milk production traits in our samples.

**Conclusions:**

Taken together, our findings strongly indicate that *UGDH* gene could be involved in genetic variation underlying the QTL for milk production traits.

## Background

Both theoretical and simulation studies agree that application of gene-assisted selection has the potential to increase the rate of genetic gain by pre-selecting young candidate bulls prior to progeny testing in dairy cattle [1]. This kind of selection is based on the identification of genes that may affect the traits of interest. In dairy cattle, since the first report on quantitative trait locus (QTL) mapping by Georges et al. [2], extensive QTL mapping has been implemented to detect QTLs with major effect on the milk production traits. So for, a total number of 1,651 QTLs for milk yield and composition traits have been reported via genome scan based on marker-QTL linkage analysis ( http://www.animalgenome.org/QTLdb/cattle.html, April 29, 2012). With the completion of genome sequencing of cattle and development of comparative map of human/bovine, it is feasible to identify the genes or other functional elements, which are responsible for a quantitative trait with comparative candidate positional cloning strategy [3]. In recent years, based on fine mapping results and positional cloning tools, several positional candidate SNPs have been confirmed to be true QTL, including the AA to GC dinucleotide substitution (K232A) in the exon8 of *DGAT1* on BTA 14 [4] and an F279 missense mutation of *GHR* on BTA20 [5] for fat percentage, a mutation in the regulatory element of *OPN* on BTA6 [6] and a Y581S missense mutation of *ABCG2* on BTA6 [7] for milk fat and protein concentration.

Even though QTLs for milk production traits have been found in almost 29 chromosomes, BTA6 is one of the most concerned chromosomes in QTL mapping for milk yield and content. Of the 1651 reported QTLs for milk production traits, 193 are on BTA6. In our previous study in a daughter-design Chinese Holstein population consisting of 26 sire families, a QTL for milk yield, fat yield, protein yield and fat percentage was detected around the microsatellite marker *BM470* with a confidence interval of 4 cM on BTA6 by using both linear regression and variance component approaches [8,9]. This region was also found to harbor one or more QTLs for milk production traits by several previous independent studies [10-13]. Thus, further fine mapping has been employed with 15 microsatellites in the region of 14.3 cM between markers *BMS690* and *BM4528* in 918 daughters of 8 segregating families identified by Chen et al. [8,9]. With haplotype sharing based LD and single marker regression mapping, a QTL with significant effects on milk yield and milk composition was determined between *BMS483* and *MNB209*, which spans a narrow genetic distance of 0.6 cM and a physical distance of 1.5 Mb [14]. In the present study, by using a high-resolution whole-genome cattle-human comparative map [15-17] and the bovine genome assembly Btau 4.0 ( http://www.ncbi.nlm.nih.gov), a total of 15 known genes have been mapped within such narrow linkage region. Out of them, we herein focused on *UGDH* as the most plausible comparative functional candidate gene affecting milk production traits. The enzyme UGDH can convert UDP-glucose to UDP-glucuronic acid, a critical component of the glycosaminoglycans, hyaluronan, chondroitin sulfate, and heparan sulfate, thus promotes normal cellular growth, embryogenesis and adult organism physiology [18,19]. UGDH is also implicated in the progression of epithelial cancers such as colon, breast, and prostate [20-22]. The purpose of the study was to determine the genetic effect of the *UGDH* gene on milk yield and component traits in dairy cows and regulation by functional polymorphisms.

## Methods

The blood samples and frozen semen were collected along with the regular quarantine inspection of the farms and breeding station, so no ethical approval was required for this study.

### Animals and phenotypic data

A total of 1417 Chinese Holstein cows were selected from 8 sire families with 67–540 daughters in each family. Such 8 sires represent all the segregating families detected in our previous QTL mapping studies [8,9,14]. Estimated breeding values (EBV) for five milk production traits (i.e., milk yield, fat yield, protein yield, fat percentage, and protein percentage over 305 days) were provided by the Dairy Data Processing Center, Dairy Association of China (DAC) which was calculated with a random regression multiple traits test-day model based on 6,980,000 test-day records of 585,121 Holstein cows collected from 1993 to 2008 in China.

Genomic DNA was extracted from whole blood samples of cows and frozen semen of the 8 bulls by a standard phenol-chloroform method and stored at −20°C.

### Positional candidate cloning, SNP discovery and genotyping

Within the 1.5-Mb region between microsatellites *BMS483* and *MNB-209* on BTA 6 where harbored a QTL for milk production traits [8,9,14], positional candidate cloning was implemented to mine the known functional genes by bioinformatic analysis of a high-resolution whole-genome cattle-human comparative map [15-17] and the bovine genome assembly Btau 4.0 ( http://www.ncbi.nlm.nih.gov). Then, based on Btau 4.0, full-length cDNA sequences of candidate genes were obtained and totally 88 pairs of primers were designed with Primer3.0 program to amplify the entire coding region of each candidate gene to identify potential polymorphisms (primers were not shown with the exception of *UGDH*).

Twelve pairs of primers for amplification of all the 12 exons and partial introns of *UGDH* were presented in Table 1. Pooled DNA from the 8 bulls was amplified at each exon followed by directly sequencing the PCR products to search SNPs. DNAMAN 6.0 (Lynnon Biosoft, USA) and Chromas 2.0 (Technelysium, Australia) were run for alignment between sequences of the 8 bulls and the reference sequences in NCBI. Individual genotyping of the discovered SNPs was performed for all of the 1417 cows with SNPlex assay (ABI, USA) or TaqMan probe (ABI, USA). For the 8 bulls, SNP genotyping was performed by direct sequencing.

**Table 1:** **PCR primers for amplification and identification of single nucleotide polymorphisms in *UGDH***

**Amplicon number**	**Amplicon content**	**Forward primer sequence**	**Reverse primer sequence**	**Amplicon size (bp)**
1	Exon 1	GAA TGA ATC CTC CGC CT	TTCCTG TGG TGA TGT TCG	437
2	Exon 2	CTTTCCCTATACCTGTCACC	ACTCGTCATCTCACTCCACT	508
3	Exon 3	GTTCAGTTGACCTGCTGCTC	CTAGGTGCTCAAGTGTGCTC	443
4	Exon 4	GGG CTA CTG ATG ATG TT	CTC TGT CTG GGT TCT TC	494
5	Exon 5	AGC AGA AAG TAT TCG TCG	CAG CTT AGC AGC AGG TaG	450
6	Exon 6	ACC AAC CCA CCC ATT GCC T	CGG ACA CGA CTG AGC TAC T	669
7	Exon 7	AGT TGA TTA GTT TCT TGC	TAA TAA TTG AGT TCC TGG	744
8	Exon 8	TTT GGA GAA GTT GGC TAA GG	TGC TAT GCT TCT GAA TGG C	707
9	Exon 9	CGT TCA TAT CCT CAC AAG A	TGG GGA AAT CGT TAC AGT A	522
10	Exon 10	TTC AAC CAG ACC ATT CCA CT	TGC GTT CAT AAT CCA GTT CC	339
11	Exon 11	TAA GGT GAA GTG ACG GAA GC	GGA GCA ACT AAG CCT GTG TG	473
12	Exon 12	GGG AATGTA GAC TGA CTC	GCT ACT CAT CCA ATC ACT	1801

### Association analysis

#### Single SNP analysis

Associations between individual SNP and the five milk production traits were estimated using the MIXED procedure of the SAS 8.02 software based on the model *y* = *μ* + *G* + *a* + *e*, where y is the EBV of cows, *μ* is the general mean, *G* is the SNP genotype effect, *a* is the random polygenic effect with distribution of N(0, **A** [*σ*_*a*_^2^]) (**A** is the additive genetic relationship matrix among all individuals, *σ*_*a*_^2^ is the additive genetic variance), and *e* is a random residual with distribution of N(0, **W***σ*_*a*_^2^) (**W** is a diagonal matrix with the diagonal elements equal to 1/*REL*_*ij*_ , *REL*_*ij*_ is the reliability of the EBV of daughter j in family i, and *σ*_*a*_^2^ is the residual error variance).

### Haplotype analysis

Pair-wise linkage disequilibrium (LD) between all SNP pairs and haplotype blocks were estimated using the program Haploview4.0 [23]. Then, haplotype reconstruction of each cow was carried out within the blocks via expectation maximization (EM) algorithm [24]. Association between each haplotype and the five milk production traits was performed based on the model *y*_*ij*_ = *μ* + *s*_*i*_ + *bh*_*ij*_ + *e*_*ij*_, where *s*_*i*_ is the fixed effect of sire *i*, *b* is the regression coefficient, hij is an indicator variable with a value 0, 1 or 2 to indicate the copy number of the haplotype carried by the individual.

Both analyses (e.g. Single SNP analysis and Haplotype analysis) were corrected for multiple testing using false discroverage rate (FDR) methods. We declared a significant SNP or haplotype if the corresponding FDR value < 0.05.

### cDNA synthesis, plasmid construction and mutagenesis

On the basis of association analysis results and the nucleotide location of each SNP within the gene, we investigated three SNPs with two located in exon 1 (SNPs exon1-1 and exon1-2), one in exon 11 (SNP exon11-1). The complete coding region of UGDH was synthesized based on the sequence of cattle full-length cDNA at NCBI (GenBank Accession No. NM: 174211). To make cloning step easier, we added a restriction endonuclease enzyme digestion site to the 5^′^ and 3^′^end of the synthesized gene, respectively. The synthesized DNA fragment was double-digested with restriction enzymes *Bam*H I and *Xho* I and then cloned into the digested pcDNA3.1/myc-HisA expression vector (Clontech, Mountain View, CA) with the same enzymes; this yielded the first plasmid construct, named as pcDNA3-GAC (g-a-c). The other seven plasmid constructs (i.e., a-a-c, g-c-c, g-a-t, a-c-c, g-c-t, a-a-t, and a-c-t) were generated from the g-a-c plasmid using a site-directed mutagenesis kit purchased from Stratagene Inc. (La Jolla, CA). All eight plasmid constructs were verified by automated DNA sequencing in both directions.

### Cell culture and transfection

COS-7 cells (an African Green Monkey SV40-transf'd kidney fibroblast cell line) were purchased from the American Tissue Cell Culture Inc. (Manassas, VA), and cultured in RPMI-1640 supplemented with 10% fetal bovine serum (Invitrogen, Carlsbad, CA) at 37°C in 5% CO_2_. The culture medium was changed every other day. The cells were transfected by Lipofectamine 2000 (Invitrogen, Carlsbad, CA) according to the manufacturer’s protocol. Briefly, a day prior to transfection, the cells were trypsinized and plated at 4 × 10^4^ cells per well on a 48-well plate. Subsequently, 0.4 μg of plasmid DNA containing the appropriate structure was diluted in 20 μl of Opti-MEM I (Invitrogen, Carlsbad, CA) Reduced Serum Medium and mixed with an equal volume of Opti-MEM I Reduced Serum Medium containing 1 μl of Lipofectamine 2000. The mixture was incubated for 20 min at room temperature and added directly to the cells in a 48-well plate. The cells were cultured for an additional 48 hours prior to harvesting for extraction of RNA and protein for real-time RT-PCR and Western blotting, respectively.

### RNA isolation and real-time quantitative RT-PCR

To evaluate the regulatory effect of SNPs exon1-1 (G/A), exon1-2 (A/C) and exon11-1 (C/T) on the expression of UGDH, eight plasmids were constructed corresponding to different allele combinations of exon1-1 (G/A), exon1-2 (A/C) or exon11-1 (C/T). Total RNA from cell transfected by eight plasmids respectively and control cells was extracted using TRIzol reagent (Invitrogen, Carlsbad, CA). One microgram of total RNA was reverse transcribed in a final volume of 20 ul containing 4 ul of 5 first-strand buffer (250 mM Tris–HCl, pH 8.3; 375 mM KCl; 15 mM MgCl2), 10 mM DTT, 0.5 mM each dNTP, 40 U RNaseOUT™, 1 ul of 50 nM random hexamers, and 200 U of Superscript II RNase H- reverse transcriptase (Invitrogen). The TaqMan® specific probes and primers used for real-time PCR were designed according to the above synthesized UGDH fragment and myc sequences in pcDNA3.1/myc-HisA expression vector were: forward primer 5′-CTCCTTCTGGTGAAATTCCAAAGT-3′, reverse primer 5′-TCTAGACTCGAGTACTCTGGGTTTCTT-3′, and the probe 5′-AGTCTTCAGGATATGTGCCCAA-3′. Amplification of 2 ul of cDNA was carried out in a total volume of 20 ul according to the manual of TaqMan® Gene Expression Assays (Applied Biosystems). The mRNA level of each gene was determined using a calibration method [25] and normalized to that of 18S rRNA in each sample. In addition, the RNA of transfected cells was treated with RNA-free DNase I (Ambion Inc, Foster City, CA) to remove any potential remaining plasmid DNA from the RNA sample prior to reverse transcription. All the primers and probe were synthesized by Applied Biosystems Inc. (Foster, CA).

### Western blot

Total protein was extracted from individual transfected cell from an independent experiment by homogenization in RIPA buffer (50 mM Tris–HCl, pH 8.0; 150 mM NaCl, 1% NP-40, 0.5% sodium deoxycholate, and 0.1% SDS), and the protein concentration was determined by the Bio-Rad Protein Assay (Bio-Rad Laboratories, Hercules, CA). Ten micrograms of total protein was separated by 10% SDS-PAGE followed by transfer to nitrocellulose membranes (0.45 um) at 25V overnight at 4°C. The membrane was first incubated in a blocking buffer (5% nonfat milk and 0.2% Tween 20) for 1.5h at room temperature and then 1.5h at room temperature in the blocking buffer containing cattle anti-His antibody (dilution 1:5000; Abcam Inc, Cambridge, MA). After three washes in TBST (10 mM Tris–HCl, pH 8.0; 0.15 M NaCl; 0.2% Tween 20) for 10 min each, the membranes were exposed to horseradish peroxidase-conjugated secondary antibody at room temperature for 1.5h, and then exposed to X-ray film. After hybridization with the antibody of interest, membranes were stripped and re-probed with antibody to β-Actin (dilution 1:5000; Abcam Inc, Cambridge, MA), which was used for normalization of the protein content of each sample. Then, the films were scanned for quantitative analysis with ImageQuant 5.2 (Molecular Dynamics, Sunnyvale, CA).

## Results

### Positional candidate cloning

With bioinformatic analysis of whole-genome cattle-human comparative map and the bovine genome assembly Btau 4.0, totally 13 annotated genes and 2 putative genes were mapped within the previously identified 1.5Mb linkage region on BTA6 (Mei et al., 2009), which were compose of *TBC1D1*, *KLF3*, *TLR10*, *TLR1*, *TLR6*, *LOC511583*, *TMEM156*, *LOC528668*, *WDR19*, *LOC514842*, *LOC514925*, *RPL9*, *LIAS* and *UGDH*. Of them, five genes including *KLF3*, *KLHL5*, *RFC1*, *WDR19* and *UGDH*, were suggested as candidates for milk production traits based on their corresponding physiological and biochemical functions in human, so that the entire coding region of each gene was screened to identify any potential polymorphisms by direct sequencing of pooled DNA from 8 segregating sire families. As a result, a total of 64 SNPs were revealed and their associations with five milk yield and milk composition traits were determined (data of *KLF3, KLHL5, RFC1 and WDR19* were not shown). We herein considered *UGDH* as the most plausible comparative functional candidate gene affecting milk production traits.

### SNPs identification and selection

**Figure 1:**
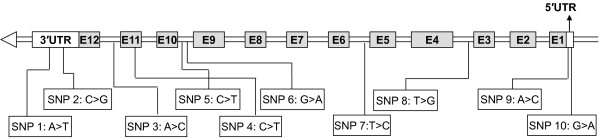
**Location of 10 identified SNPs in *UGDH***.

As for *UGDH* gene, a total of 22 SNPs, including 12 in exons and 10 in introns, were found from the pooled DNA of the 8 unrelated bulls. 10 SNPs out of them were selected for the association analysis according to their heterozygosities among the 8 bulls and locations (polymorphisms in exons were preferred, Figure 1). The locations and allele frequencies of the 10 SNPs are shown in Table 2.

**Table 2:** **Information for 10 SNPs in *UGDH *and allele frequencies in Chinese Holstein population**

**SNP number**	**SNP name**	**SNP location**	**Position (BTA 6)**	**Alleles**	**Frequency**	**Primer and probe sequence**
1	SNP Ex12-3	Exon 12	60966191	A/T	0.40/0.60	F: ATTTCTCGTCCCCCTGAATTGAG
						R: ACGTGGCAGCATAGACAGAAAA
						P: AAGGCCACACACTGT-MGB
2	SNP Ex12-2	Exon 12	60966411	C/G	0.42/0.58	F: GTACTCTTGCATGGGAAGTTCTACA
						R: CGCTAAGTTGTGTCTGACTCTGTTA
						P: ACTGTAGCCCGCCAAG-MGB
3	SNP Int11-1	Intron 11	60969505	A/C	0.39/0.61	CAAACAAATG [A/C] AACAAAACCA
4	SNP Ex11-1	Exon 11	60969627	C/T	0.66/0.34	F: CCTGTATATGCTCTAATTATTTCTCTGGTTCTT
						R: CGTCAAAGATAAAGGCTGGTTTCAG
						P: CATTTTTTTATGAATACGTTCAT-MGB
5	SNP Int9-3	Intron 9	60969880	C/T	0.59/0.41	AAATCACTCC [C/T] CTCTTACTCC
6	SNP Int9-2	Intron 9	60969929	G/A	0.46/0.54	TCAGACTCTC [G/A] ATTCCTCCTG
7	SNP Int5-1	intron5	60975997	T/C	0.91/0.09	GAGAAATAAG [T/C] CCTTGGCTTA
8	SNP Inti3-1	Intron 3	60979545	T/C	0.32/0.68	AGCCATAGCA [T/G] TACTTAGTAT
9	SNP Ex1-2	Exon 1	61000215	A/C	0.75/0.25	TGGTTGCAGC [A/C] CTCGGTGCCT
10	SNP Ex1-1	Exon 1	61000233	G/A	0.09/0.91	GAGCCGAGGA [G/A] ATAGAAGTGG

Note:

Genotyping platform: ABI7900, SNP 1, 2, 4; Mass Spec, SNPs 3, 5–10.

### Association between individual SNP and milk production traits

Single-locus association analysis (Table 3) showed that three SNPs (SNP Ex1-1, SNP Int3-1 and SNP Ex12-3) were significantly associated with protein yield with their raw P values < 0.05 through single SNP analysis, and 2 of them (SNP Ex1-1 and SNP Ex12-3) also associated with milk yield. However, only the association (SNP Ex1-1 with milk yield) remained significant after correction for multiple testing (P = 0.0003).

**Table 3:** **Significant *UGDH *SNPs associated with milk production traits in Chinese Holstein population (P<0.05)**

**SNP number**	**SNP**	**Genotype**	**Milk yield, kg LSMEAN ± SE**	**Protein yield, kg LSMEAN ± SE**
1	SNP Ex12-3	AA	168.55±230.51	3.99±6.33
		AT	275.43±160.02	9.93±4.39
		TT	514.41±172.42	15.11±4.73
8	SNP Int3_1	GG	157.24**±**319.55	5.97±8.80
		TG	279.02**±**180.27	8.61±4.94
		TT	380.43**±**180.47	14.62±4.95
10	SNP Ex1-1	AA	−692.88±745.73*	−12.54±20.55
		GA	−4.3120±199.98*	2.01±5.49
		GG	467.51±157.89*	13.08±4.33

Note:

* Significant at FDR<0.05.

### Associations between haplotypes and milk production traits

**Figure 2:**
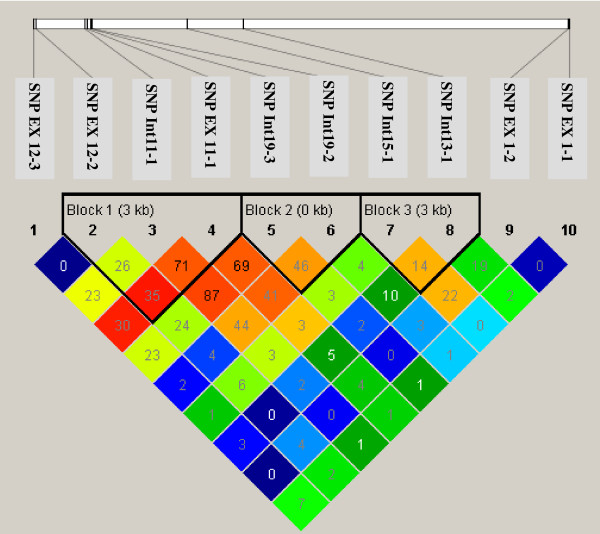
**Haplotype block of 10 identified SNPs in *UGDH***.

Figure 2 shows pair-wise D′ values and predicted haplotype blocks for the ten SNPs. Three blocks were identified. The first block consisted of 3 SNPs which formed 7 haplotypes in the studied population. The other two blocks both consisted of 2 SNPs which formed 4 haplotypes (Table 4). As shown in Table 4, three major haplotypes showed a significant association with two milk _percentage traits_. The haplotypes G-C-C, formed by SNP Ex12-2, SNP Int11-1, and SNP Ex11-1, showed a significant association with _protein percentage_ (F = 4.15; P = 0.0418). The second haplotype, T-G, formed by SNP Int9-3 and SNP Int9-2, was significantly associated with _fat percentage_ (F = 5.18; P = 0.0231). The last one, C-C, formed by SNP Int5-1 and SNP Int3-1, exhibited a significant association with _fat percentage_ also (F = 7.25; P = 0.0072) which remained significant after multiple testing correction of FDR (adjusted significance level was 0.0083 for four major haplotypes).

**Table 4:** **Associations of *UGDH *haplotypes with milk production traits in Chinese Holstein population**

**2**	**3**	**4**	**5**	**6**	**7**	**8**	**Freq**	**Milk yield**		**Protein yield**		**Fat percentage**		**Protein percentage**	
								_**F**_**value**	**P value**	_**F**_**value**	**P value**	_**F**_**value**	**P value**	_**F**_**value**	**P value**
_G_	_A_	_C_					0.1993	0.07	0.7947	0.20	0.6540	2.11	0.1468	1.45	0.2294
_G_	C	_T_					0.3802	0.24	0.6226	0.14	0.7084	0.42	0.5183	0.24	0.6263
_G_	_C_	_C_					0.1253	2.7	0.106	2.33	0.1272	2.03	0.1543	4.15	**0.0418**
			_T_	_G_			0.4282	0.77	0.3803	0.60	0.4404	5.18	**0.0231**	0.19	0.6601
			C	_G_			0.1390	0.39	0.5324	0.00	0.9526	0.14	0.7060	1.71	0.1907
			C	_A_			0.4229	0.03	0.8666	0.09	0.7590	0.24	0.6218	0.82	0.3659
					_T_	_T_	0.7441	1.52	0.2185	1.77	0.1836	7.25	0.0072*	0.05	0.8237
					T	G	0.1620	0.33	0.5660	0.04	0.8373	0.00	0.9987	0.72	0.3954

Notes:

1) Only major haplotypes with a frequency >0.10 are shown.

2) * Significant at FDR<0.05.

Analysis of *UGDH* and its mutant Ex1-1 (G/A), Ex1-2 (A/C) and Ex11-1 (C/T).

**Figure 3:**
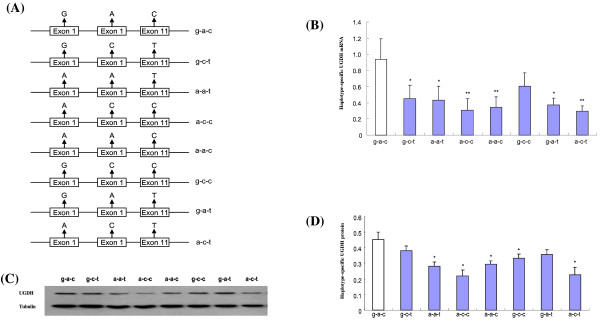
**Expression analysis of all combinations of SNPs Ex 1–1, Ex 1–2 and Ex 11–1.** (**A**) Illustration of eight haplotype constructs for *UGDH*. (**B**) Statistical analysis of real-time RT-PCR data for the eight plasmid constructs. 18sRNA was used to normalize expression of each construct. Compared with g-a-c, g-c-t, a-a-t, a-c-c, a-a-c, g-a-t, and a-c-t decrease the expression of *UGDH*, by 51.8%, 53.9%, 61.6%, 63%, 59.7% and 68.2%, respectively. (**C**) Representative Western blotting images for eight constructs of *UGDH* and β-Actin. (**D**) Statistical analysis of the protein level of eight constructs of *UGDH* normalized to β-Actin, which indicated a-a-t, a-c-c, a-a-c, g-c-c, and a-c-t similarly decreased UGDH protein expression, by 37.8%, 51.1%, 34.8%, 26.6%, and 50.1%, respectively, compared with the construct g-a-c. Values are given as means ± S.E.M. (* P <0.05, ** P <0.01; n = 5/group.

To define the functional significance of SNPs Ex1-1 (G/A), Ex1-2 (A/C) and Ex11-1 (C/T) on the expression of *UGDH*, we created plasmid constructs for the eight possible combinations (i.e., g-a-c, a-a-c, g-c-c, g-a-t, a-c-c, g-c-t, a-a-t, and a-c-t) by site-directed mutagenesis from a complete *UGDH* cDNA clone (g-a-c) and then transfected them into COS 7 cells. Following transit transfection, we measured the mRNA and protein expressions from each construct using real-time RT-PCR and Western blotting (Figure 3).

After normalization to the corresponding 18S rRNA of each sample, we found all mutant constructs except g-c-c showed significantly lower mRNA expression than the construct g-a-c (~52.0%; P < 0.05). Moreover, three mutant constructs (i.e., a-a-c, a-c-c, and a-c-t) were observed to be highly significant (P < 0.01), where *UGDH* mRNA was down-regulated by 63%, 62%, and 68%, respectively (Figure 3B). At the protein level, as shown in Figure 3D, the seven mutant constructs showed generally lower expression than the construct g-a-c (−15.8~−51.1%) and among them, constructs a-a-t, a-c-c, a-a-c, g-c-c, and a-c-t reached the significance level of 0.05, with their UGDH protein concentration being reduced by 37.8%,51.1%, 34.7%, 26.5%, and 50.1%, respectively.

These results indicate that at mRNA level, the mutation of Ex1-2 has little impact on the expression of *UGDH* and that all differences observed among the eight constructs were attributable primarily to SNPs Ex1-1 and Ex11-1, for which the A allele of SNP Ex1-1 and T allele of SNP Ex11-1 caused lower expression than the G and C allele respectively. But at protein level, the two alleles of SNP Ex1-1 play more important role in the expression of *UGDH* than those of SNPs Ex1-2 and Ex11-1, where the G allele of SNP Ex1-1 corresponds to higher protein concentration.

## Discussion

In this study, we provide a strong evidence for the significant associations of *UGDH*, with 2 milk production traits in a Chinese Holstein cattle population. Moreover, we demonstrated that the SNPs Ex1-1 and Ex11-1 in exonic regions of *UGDH* are two functional polymorphisms in which A allele of SNP Ex1-1 and T allele of SNP Ex11-1 cause lower expression level of *UGDH* compared with the G and C allele respectively. Together, our findings not only confirmed our early finding that a QTL located in the 1.5-Mb region between *BMS483* and *MNB-209* for milk yield and protein yield [14], but also strongly suggest that the SNPs Ex1-1 and Ex11-1 in *UGDH* might be QTN responsible for this QTL.

Previously, we mapped a QTL near BMS470 with effects on milk production traits with daughter design using 14 microsatellites on bovine chromosome 6 in a Chinese Holstein population involving 26 paternal half-sib families with 2356 daughters [8,9]. By increasing the marker density with 17 microsatellite markers spanning from *BMS690* to *BMS4528* near *BMS470* and genotyping them for 918 daughters from 8 sires families which had been proved to contain segregating QTLs affecting milk production traits in the previous studies [8,9], we further fine mapped this QTL in region of BMS483 and *MNB-209* by employing linkage disequilibrium (LD) mapping approaches [14]. On the base of these studies, the *UGDH* gene within this interval was selected as a potential positional candidate gene for further study by genetic association and functional analysis as reported in this study. Association analysis of individual SNPs indicated that the SNP Ex1-1 located in the 5` UTR (c.exon1-1 G>A) was significantly associated, even after multiple testing correction of FDR, with milk yield (P = 0.003). Moreover, haplotype analysis revealed two significant associations of two percentage traits with haplotypes formed by SNP Ex12-2- SNP Int11-1- SNP Ex11-1 and SNP Int5-1- SNP Int3-1, respectively. Given the aforementioned association results of SNP Ex1-1 and SNP Ex11-1 with milk production traits and their locations within the gene, we hypothesized that these two SNPs and the other SNP Ex1-2 (located in the 5` UTR of *UGDH* and near to the SNP Ex1-1) contribute to the observed association of *UGDH* with milk production traits. By expressing plasmid constructs containing different alleles for above three SNPs in COS 7 cells, we found that the plasmid construct containing the A allele of SNP Ex1-1 and T allele of SNP Ex11-1 produced significantly lower expression of mRNA than the G and C allele respectively. At protein level, the two alleles of SNP Ex1-1 play more important role on the expression of *UGDH* than those of SNPs Ex1-2 and SNP Ex11-1, where the G allele of SNP Ex1-1 corresponds to higher protein concentration. Thus, we concluded that SNP Ex1-1 is involved in the regulation of the expression both at the transcription and protein level and SNP Ex11-1 is involved in the regulation of the expression of *UGDH* at the transcription level Although further study is required to determine how these two DNA polymorphisms modulates the expression of *UGDH*, they are most likely responsible, at least part, for the observed effect of above QTL affecting milk yield and composition in dairy cattle used in current study. As for SNP Ex1-2, in agreement with the results of the individual association analysis on this SNP, we did find no significant difference between the two alleles at either the RNA or the protein level. Although a non-synonymous SNP in a gene can change its function(s), causing the gene to be associated with a disease [26], some recent studies found that synonymous SNPs without altered coding sequences also can affect the gene’s function. These SNPs may alter the structure of the substrate and inhibitor interaction site by a rare codon in their polymorphism [27] or affect gene expression by altering mRNA secondary structure [28].

## Conclusions

Based on the previously identified 1.5-Mb region between markers *BMS483* and *MNB-209* on BTA6 including a QTL for milk production traits, the *UGDH* gene was identified and shown to be associated milk yield and milk composition. Further *in vitro* expression assay demonstrated that the SNPs in exon1 and exon11 of *UGDH* represent two functional polymorphisms affecting expression of *UGDH* and might partly contributed to the observed association with milk production traits.

## Abbreviations

ABCG2: *ATP*-binding cassette; BTA: Bos Taurus Automosome; DAC: Dairy association of China; DGAT1: AcylCoA: diacylglycerol acyltransferase; GHR: Growth hormone receptor; LD: Linkage disequilibrium; SNP: Single nucleotide polymorphism; OPN: Osteopontin; QTL: Quantitative trait locus; UGDH: UDP-glucose dehydrogenase.

## Competing interests

The authors declare that they have no competing interests.

## Authors’ contributions

QX performed the cell related experiments and prepared the manuscript. GM contributed to the SNP discovery and genotyping and association analysis. DS conceived and designed the experiments and prepared the manuscript. QZ and YZ participated in the experiment design and result interpretation. CY and HC performed the QTL mapping. XD and JL participated in the data analysis. All authors read and approved the final manuscript.
